# Cost-effectiveness analysis of fractional flow reserve versus angiography among patients with coronary artery disease undergoing borderline coronary lesions treatment in Iran

**DOI:** 10.1186/s12962-022-00402-y

**Published:** 2022-12-08

**Authors:** Khosro Keshavarz, Rita Rezaee, Elahe Esmaili, Roohollah Mansouri, Abdosaleh Jafari, Azadeh Erami, Hamid Talebianpour, Ricardo Fonseca, Mozhgan Fardid

**Affiliations:** 1 Health Human Resources Research Center, School of Health Management and Information Sciences, Shiraz University of Medical Sciences, Shiraz, Iran; 2 Clinical Skill Lab Center, Shiraz University of Medical Sciences, Shiraz, Iran; 3 Shiraz alzahra hospital, Shiraz University of Medical Sciences, Shiraz, Iran; 4 Student Research Committee, Tehran University Of Medical Sciences, Tehran, Iran; 5 Menzies Institute for Medical Research, University of Tasmania, Hobart, Australia; 6 Shiraz University of Medical Sciences, Shiraz, Iran

**Keywords:** Cost-effectiveness analysis, Fractional flow reserve (FFR), Angiography, Coronary artery disease

## Abstract

**Background:**

The present study aimed to examine the cost-effectiveness of fractional flow reserve (FFR) versus angiography in treating borderline coronary lesions in patients with coronary artery stenosis in Iran. Cardiovascular disease is a leading cause of morbidity, mortality, readmission and the most important cause of disability in many countries, including Iran.

**Methods:**

This was a cost-effectiveness study conducted from the perspective of the Ministry of Health in 2019. The effectiveness was determined using four indicators: Quality Adjusted Life Years (QALYs), major adverse cardiac events (MACE), angina, and number of used stents (mean). Only direct medical costs (DMC) were estimated. To evaluate the cost-effectiveness of FFR versus angiography, A decision tree model was built by patient’s level data.To coping with uncertainty Probabilistic sensitivity analysis (PSA) was performed.

**Results:**

Totally, 98 cases of FFR and 238 cases of angiography were included in the analysis. The average of QALY in FFR and angiography were 0.853 and 0.787, respectively. The cost of these methods were $6128 and $8388, correspondingly. Therefore, FFR was dominant compared to angiography. Results of the scatter plots and acceptability curve showed that FFR was more cost-effective than angiography in 94% and 96% of simulations for a threshold lower than $11,000 PPP. The PSA analysis confirmed the robustness of the study results.

**Conclusion:**

The results indicated that FFR was more cost-effective than angiography in the cases studied in Iran. Consequently, FFR can be used as a high-priority diagnostic method and it is recommendable to be included in insurance coverage.

**Supplementary Information:**

The online version contains supplementary material available at 10.1186/s12962-022-00402-y.

## Background

Cardiovascular disease is a major cause of death, disease, readmission and disability in many countries of the world, including Iran [1]. Studies in Iran showed that coronary artery disease was presented in 847,470 people in 2005, and it is estimated to be doubled in 2025 [2]. The incidence of stenosis in the coronary arteries, which are the main feeding arteries of the heart, occurs in the childhood and adolescence by a plaque creation due to the lack of knowledge on the factors contributing to the formation of atherosclerosis (atherosclerosis). Then, this plaque develops in middle-age and leads to a reduction of heart feeding, chest pain and breath shortness [3]. Coronary artery stenosis is managed by several methods, including open surgery, coronary artery bypass grafting, and angioplasty (PCI) [3]. In general, angioplasty complications can be categorized as follows: During angioplasty, blood flow may be blocked which is due to the substances released from the stent into the bloodstream [4]. Restraint in 30 to 40% of cases is a complication of balloon angioplasty [5]. The heart springs are designed to reduce restenosis [6]. Genuine pure metal stents, reduce the chance of restenosis by up to 20% while pharmaceutical stents reduce it by less than 10% [7]. The diagnostic and therapeutic procedure is an ambiguity of interventional cardiology for those with coronary stenosis whose severity cannot be clearly stated. FFR (Fraction Flow Reserve) may significantly meet such needs. It is an accurate method for measurement of coronary stenosis which is less invasive in comparison to angiography [8].

Knowing the precise cost of FFR as a valuable tool in assessing coronary stenosis among patients with functional significance of coronary artery stenosis (CAD) helps to select the appropriate treatment. Studies in this area, including Imani et al. (2015) on the cost of cardiac patients in Tabriz, showed that the total cost of cardiac patients was $1021. Seo et al. showed that the total cost of heart disease in Korea was $1 billion. Global estimates also indicate that the total cost of heart failure patients in the US will be increased from $31 billion in 2012 to $70 billion by 2030. Given necessity of further investigation for optimal resources allocation in the health sector, this study aimed to evaluate the cost-effectiveness of FFR diagnostic procedure compared to angiography in several centers in Iran [9]. Considering the importance of this disease and its imposed costs on households, and also the limited knowledge about its effectiveness as well as the necessity of conducting an economic evaluation of the FFR diagnostic procedure, this study was conducted in Iran.

## Methods

### Study design

This study was conducted in the Shahid Faghihi and Kosar hospitals in Shiraz, and the Shahid Rajaee Hospital in Tehran, Iran in 2019. These hospitals are the referral centers for FFR. Patients who had undergone angiography within the last 6 months were included in the study. Patients who did not respond to the questions or were not willing to participate, were excluded. In total, one hundred patients were allocated to FFR group and 240 to the angiographic diagnostic group as the sample size.

### Treatment costs

Required data for this study were divided into two sections: cost and effectiveness. Costs related to coronary artery disease were identified and measured from the perspective of the Ministry of Health. These items included only direct medical costs (DMC) and resource quantities such as surgeries procedures, medications, consuming materials, visit and consultation, services of nursing and patient companion and para clinic services were estimated from the patients’ medical records and expert opinions based on the prices of 2019. To calculate costs, a bottom-up approach was applied. Components of DMC included the cost of angiography and FFR, the cost of hospitalization, surgery, physician visits, medications, and all diagnostic medical services up to study and data collection. All DMC included all clinical costs, the average use percentage of each service, and the price of each service based on the tariffs of the year 2019 was entered in a form designed by experts after checking patient’s medical records, patients’ bills, expert opinions, and negotiation with the patient.

### Clinical inputs

Based on experts’ opinions and literature review, effectiveness was assessed using four indicators including Quality Adjusted Life Years (QALYs), major adverse cardiac events (MACE), angina and the mean number of stents used in the procedures. For policy and decision-making, QALYs, the incremental cost-effectiveness ratio (ICER) and the probabilistic sensitivity analysis (PSA) were used as the main outcomes.

Utility scores were obtained using EQ-5D questionnaire by telephone contact with 336 referrals to the three hospitals. The EQ-5D questionnaire is a standard tool for measuring health outcomes which is a simple description of different dimensions of health [10]. The weights of this questionnaire for Iran were estimated by Goodarzi et al., and the utility is measured through face-to-face/telephone interviews with the patient [11]. Each of these scores yields a numerical value which represents the utility in a given state of health. Score 1 indicates the best state of health and zero indicates death. Clinical outcomes including the mean of the number of stents used, major adverse cardiac events, and episodes of angina were obtained through medical records and angioplasty. procedure reports, and personal comments from patients. MACE was defined as a combination of non-lethal myocardial infarction, revascularization, and cardiovascular death. Angina is a condition in which the patient has chest pain emerged from coronary artery problems, and the reason of thoracic angina due to is insufficient oxygen supply to the heart muscle. This pain is more felt in left chest area spreadable (mostly as unstable) to the left arm (sometimes both arms), the jaw and the middle part of the two shoulders [12].

### Model structure

**Fig. 1:**
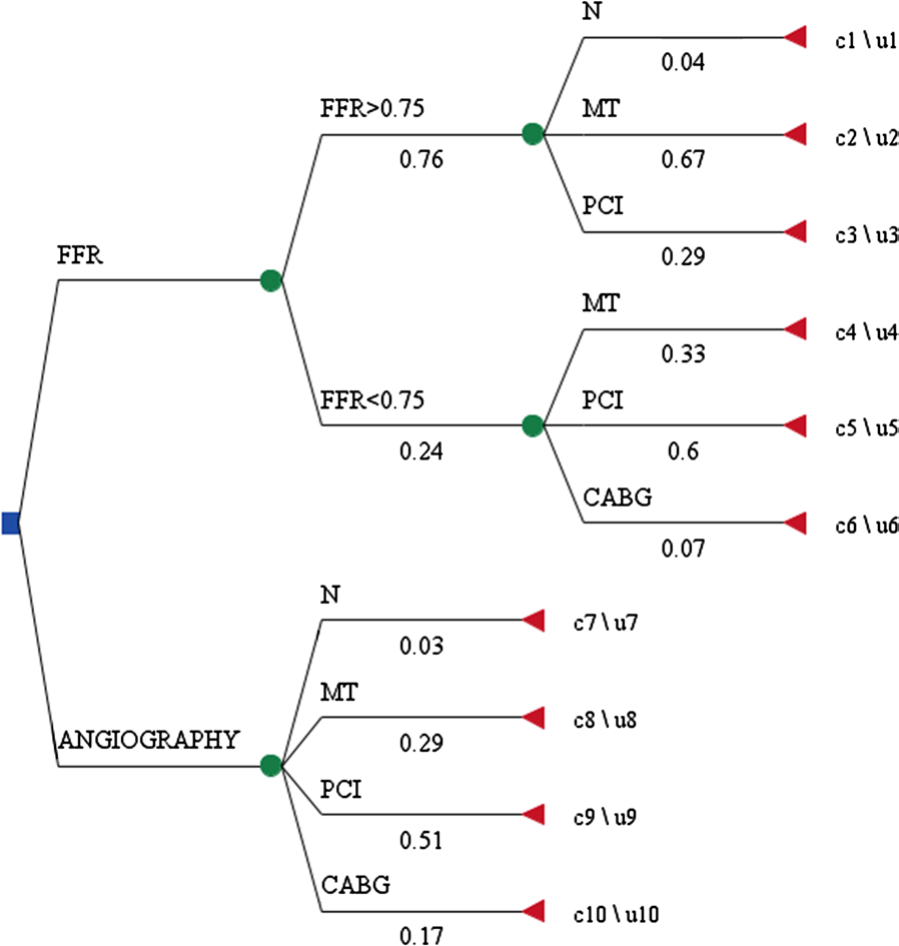
Decision tree model for FFR versus Angiography in treatment of borderline coronary lesions ***N*** normal, ***MT*** medical therapy, *PCI* percutaneous coronary intervention, *CABG* coronary artery bypass surgery, *C c*ost and U utility

To estimate the cost and effectiveness, a decision tree model was developed (Fig. 1). Costs and outcomes for each branch were identified, and the probabilities of each branch were calculated obtaining the individuals’ percentage in each treatment group after diagnosis. Patients with coronary stenosis were divided into two groups (FFR or angiography), depending on the procedure they received. FFR is also divided into two subgroups of FFR values greater than 0.75 and less than 0.75. Then, each group had three states of normal diagnosis without treatment, need for medication and angioplasty.

The present study followed the CHEERS (Consolidated Health Economic Evaluation Reporting Standard) checklist [13]. The checklist has been included in the Additional fle 1 (appendix).

### Cost-effectiveness analysis

The model was designed in TreeAge software 2011, theThe extracted data were entered into the model and the expected cost, effectiveness, and incremental cost-effectiveness ratio (ICER) were calculated. ICER was estimated using the following formula:

$$ICER=\frac{Cost A-CostB}{EffectA-EffectB}$$

### Uncertainty analysis

In this study, a PSA was performed to investigate the effects of parameter uncertainty on the model results. All variables of cost and utility were considered as a distribution. It is also worth noting that in the present study, beta (β) distributions were used to determine the probability distribution of utility values ​​that have values ​​between 0 and 1 and gamma distribution to determine the distribution of cost values. Second-order Monte Carlo simulation was performed using 5000 trials for PSA. PSA results were indicated using the cost-effectiveness acceptability curves and incremental cost-effectiveness scatter plot. An internal study by Moradi et al. was used in which the cost-effectiveness threshold for cardiac patients (2017), was at 100,767,560 Rials [14], which was adjusted for inflation rate in 2019 and estimated at $11,000 PPP.

### Statistical analysis

In this study, t-test with two independent samples was used to compare the mean of two different diagnostic methods.

## Results

Table 1 summarizes the demographic characteristics for each diagnostic group, including the number of reviewed records, age, the average length of stay in hospital and the history of hypertension, blood lipids, diabetes, heart disease, and smoking. The most common disease in patients with coronary stenosis was hypertension in both groups. The patient may have more than one of these diseases.

**Table 1:** Demographic characteristics and records of patients by treatment group

Variables	FFR		Angiography		Total	
Number of patients	98		238		336	
Age average	(60 ± 11)		(59 ± 10)		(59 ± 10.5)	
Average hospital stay days	1.3		3.5		2.85	
No Congestion of veins	6 (7%)		8 (3%)		14 (4%)	
Eclipse of a vessel	41 (42%)		71 (30%)		112 (33%)	
Eclipse of two vessels	28 (28%)		81 (34%)		109 (32%)	
Eclipse of three vessels	23 (23%)		78 (33%)		101 (31%)	
Hypertension	44%		57.1%		50.7%	
blood fat	40.3%		35.7%		38.1%	
Diabetes	32.1%		25.5%		28.9%	
History of heart disease	26.6%		21.4%		24.1%	
smoking	16.5%		5.1%		11.1%	

Table 2 shows the DMC for diagnostic procedures of FFR and angiography in dealing with the borderline coronary lesions. The results showed that the average DMC for FFR and angiography were $ 6128 and $ 8388, respectively, in which the major costs were related to the costs of surgeries (48%).

**Table 2:** The Average direct medical costs for FFR and angiography dealing with the borderline coronary lesions

Costs items	FFR (PPP$)	SE^*^	Percentage	Angiography (PPP$)	SE	Percentage
Surgeries	2941	302	48	4027	193	48
Medications	368	31	6	419	30	5
Consuming materials	1777	94	29	1426	91	17
Visit and consultation	123	12	2	755	14	9
Services of nursing and patient companion	674	54	11	1258	82	15
Para clinic	245	23	4	503	19	6
Total amount	6128		100	8388		100
Patient payment	2267		37	1678		20

* SE = standard error

### Clinical outcomes of FFR and Angiography

For two-thirds of FFR patients (64%), medication therapy was prescribed. However, for about half of angiography patients (52%), angioplasty and stent placement was applied. In both diagnostic methods, open heart surgery was rarely prescribed.

The effectiveness was determined using the incidence of major adverse cardiac events (MACE) including myocardial infarction and cardiovascular death, number of used stents, the likelihood of angina, and the utility of each diagnostic procedure were evaluated. According to Table 3, the number of used stents was 38 in FFR and 162 in the angiography group.

**Table 3:** Effectiveness data for FFR and angiography in dealing with borderline coronary lesions and the statistical relationship between clinical outcomes

	FFR	Percentage	Angiography	Percentage	T	P-Value
Number of used stents	162	–	38	–	−2.728	0.007 (0.38 − 0.061)
Average number of used stents	0.68	–	0.39	–		
Average score of health status (VAS)	0.688	–	0.782	–		
Average of utility score	0.787	–	0.853	–		
Average number of MACE	40	17	5	5	3.547	0.000 (0.182 − 0.052)
The number of angina	48	20	16	17	0.115	0.909 (0.098 − 0.078)
Mortality after at least 6 months	7	3	1	1		

Approximately 0.39 and 0.68 stents were used for each patient, respectively. The mean score of subjects from zero (death) to one (complete health) on their health status for FFR and angiography was 0.782 and 0.688, respectively based on VAS. QALYs obtained from the EQ-5D questionnaire for two mentioned diagnostic methods were 0.853 and 0.787, respectively. The more this figure is closer to one, the higher is the utility and quality of life. 5% in the FFR group and 17% in the angiography group had MACE. The incidence of angina also was 17% and 20% for FFR and angiography, respectively. The clinical outcomes of FFR and angiography were evaluated after diagnostic intervention up to the time of this study. As shown in Table 3, the mean difference in stent number between the two diagnostic groups was statistically significant (P-value < 0.01).

The upper and lower limit of the confidence interval was negative and the mean number of used stents in angiography was more than FFR. The mean difference in the number of MACEs between two groups was statistically significant (P-value < 0.001) and since the upper and lower limit of confidence interval was negative, the mean frequency of MACE in angiography is more than FFR. Furthermore, the mean frequency of angina in two diagnostic methods was not statistically significant (P = 0.91).

### Results of model estimation

The cost-effectiveness results obtained from the estimation model of decision tree are presented in Table 4.

**Table 4:** Results of estimation model of decision tree for FFR and angiography in dealing with borderline coronary lesions

Strategy name	Cost (PPP$)	QALY	Incremental cost (PPP$)	Incremental effectiveness	Result
FFR	6148	0.853	2240	− 0.065	FFR is the dominant option compared to angiography.
Angiography	8388	0.788			

The cost of each diagnostic method is obtained by multiplying the probabilities of each branch at the cost of that branch. As seen in Table 4, FFR is dominant compared with angiography due to lower cost and higher QALYs. ICER was obtained $ -34461.53 which means that for each additional QALYs FFR $ 34461.53 less cost will be imposed.

### Probabilistic sensitive analysis

**Fig. 2:**
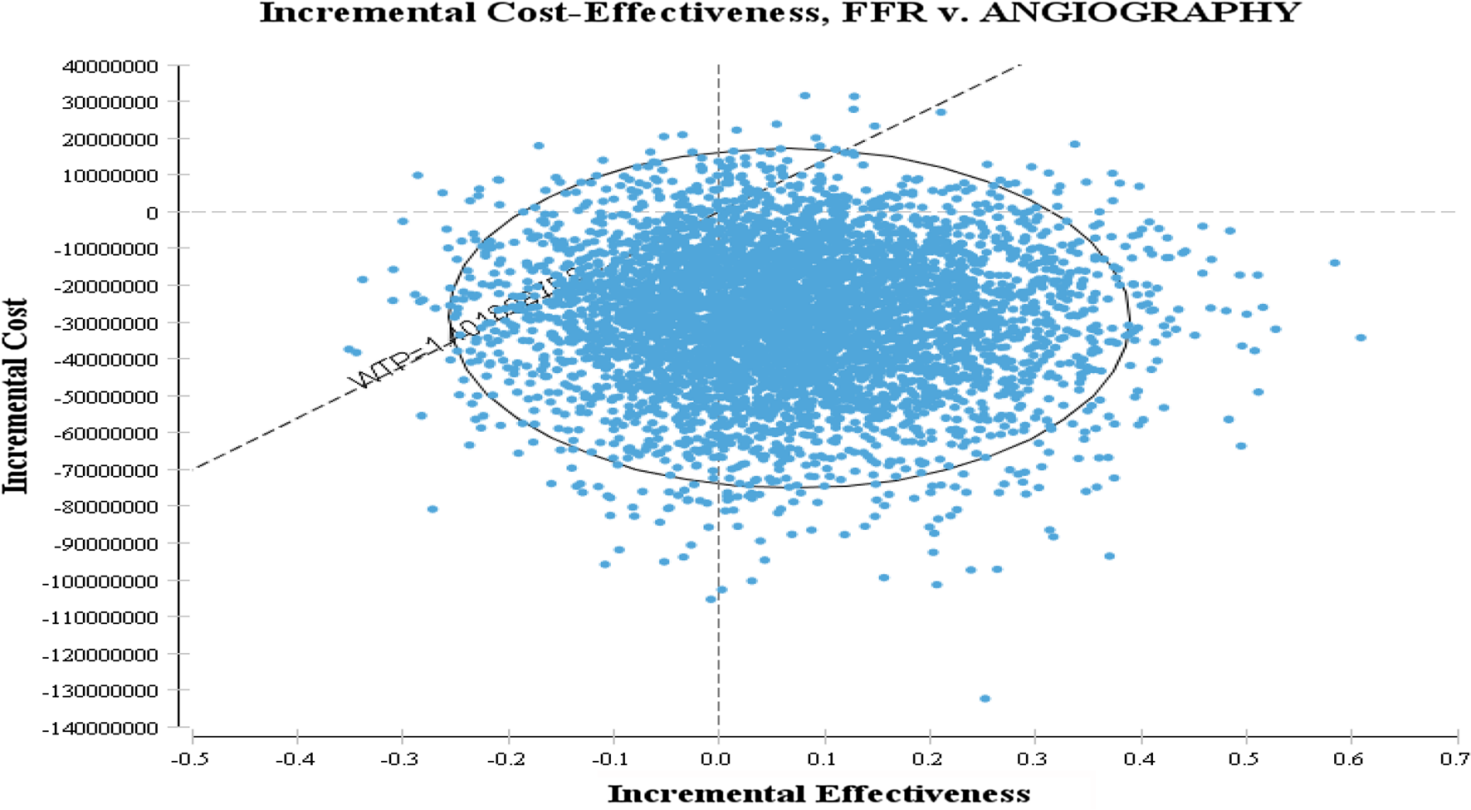
Scatter Plot of Incremental Cost-Effectiveness of FFR versus Angiography

In PSA, the parameters are determined as a distribution rather than as a single point. As shown in Fig. 2, FFR in 65% of cases is in the 4th area of the cost-effective plan, which means it is less costly and more effective than angiography and is considered as the superior strategy. In 3% of cases, it is in the one area of ​​the plan and below the threshold which has the higher cost and more effectiveness than angiography.

**Fig. 3:**
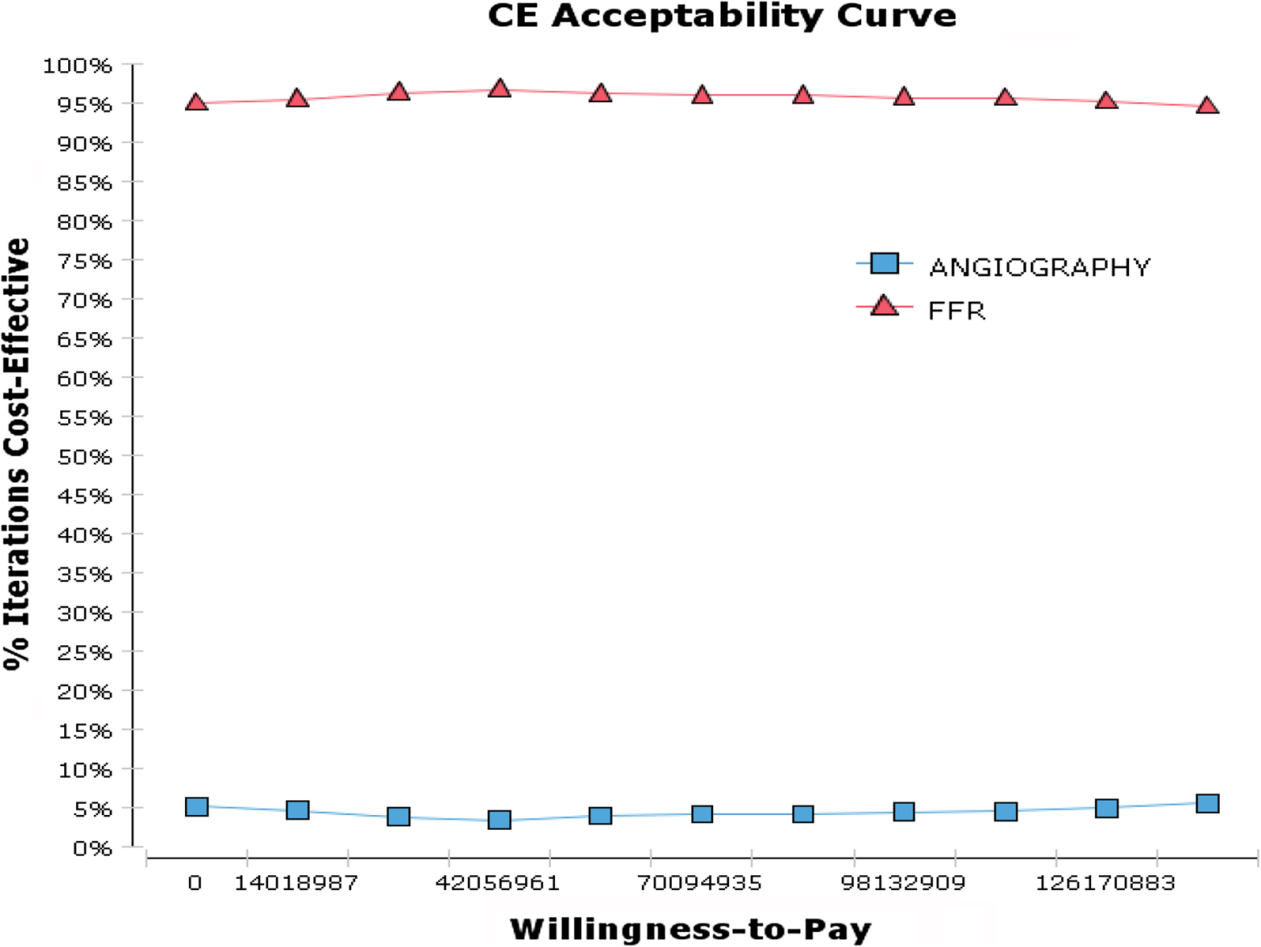
Cost-Effectiveness acceptability curve obtained from Monte Carlo Simulation for FFR versus Angiography

In 26% of cases, it is below the threshold and in the third area of ​​the plan. In other words, FFR in 94% of the cases is in the acceptance area and therefore it is the more cost-effective strategy. The cost-effectiveness acceptability curve is one of the best curves for planning and policymaking. The mentioned chart shows the percentage of bootstrapping that choose a strategy as a superior strategy. Thus, according to the results of Fig. 3, FFR is the most cost-effective treatment in 96.6% of simulations for the threshold of less than $11,000.

## Discussion

The results showed that for angiography, a higher percentage of people had the insurance of social security and health services, whereas, for FFR, this distribution was different and more than a quarter of the people had no insurance coverage, which could be the result of having no FFR coverage. In addition, the results of the current study showed that about half of people with coronary artery stenosis had hypertension. Diabetes, hyperlipidemia, a history of heart disease and smoking were other disease records of the studied subjects. Thus, the results of this study are consistent with the studies of Wilson, Turner, and many other studies that have identified these diseases as the risk factors for coronary artery stenosis [15, 16]. The cost results showed that the average cost was $6128 and $8388 for FFR and angiography respectively, in which the highest costs were related to the costs of surgeries (48%). Also, the average cost of FFR was less than angiography. Similarly, Siebert and Fearon reported lower costs for FFR than other methods [17, 18].

T The results of other clinical outcomes including the number of used stents, the incidence of cardiac adverse events and angina also showed significant differences between the mean number of used stents and the incidence of MACE in two studies’ diagnostic methods. Pim et al. showed that the mean number of stents was statistically significant. However, the mean incidence of angina in the two diagnostic methods was not significant in their study which is consistent with the results of the current study. However, the mean incidence of adverse cardiac events was not statistically significant which is inconsistent with the current study [19]. Siebert et al. in their study confirmed the effectiveness of FFR. This study showed that performing FFR about 1776 AUD saves costs and in a two-year period, the quality of life of these patients was increased ranged from 7.8 to 73.9 [18].

Shawky et al. in their study entitled showed that FFR63 intervention saved the stent in 122 lesions of 50 patients, at an average cost of ffr26000 Egyptian FFR, which was more economical than 31,000 Pound per angiography [20]. Edgard Freitas Quintella et al. (2018) revealed that Angina and resuscitation were more common on angiography than on FFR. In contrast, FFR intervention costs were higher than angiography [21].

The results of incremental cost-effectiveness ratio based on QALYs showed that FFR compared with angiography had lower cost and higher QALYs and as a result it is considered as the dominant strategy. The results of the current study are consistent with those of fearon, siebert, and kimura [18, 22, 23]. Furthermore, the results of PSA powerfully confirmed that FFR is a cost-effective alternative to angiography. This is in line with the results of William et al. on the cost-effectiveness analysis of FFR versus angiography [24].

The results of the incremental cost-effectiveness scatter plot which is another output of the probabilistic sensitivity analysis showed that FFR compared to angiography is mostly in acceptance area and below the threshold cases. Totally, FFR was in 94% of the cases in the acceptance area and below the threshold compared to angiography and thus it was considered as a more cost-effective strategy. The results of the cost-effectiveness acceptability curve, which is the output curve of the probabilistic sensitivity analysis also showed that FFR is the most cost-effective treatment in 94.5–96.6% of cases for a threshold lower than $11,000 PPP.

One of the limitations of the study was the low number of FFR patient records in Shiraz hospitals and regarding HTA’s emphasis on the examination of at least 100 cases of FFR, some of FFR cases were collected from Shahid Rajaee Heart Hospital in Tehran. Furtheremore, given that in the present study data was collected from several main reference centers of the country, the generalizability of the results would be great within the country.

And it is suggested that future studies be conducted with prospective design, larger sample size and a longer follow-up period.

## Conclusion

To sum up, according to the results of the current study it can be concluded that according to incremental cost-effectiveness ratio, MACE and the number of saved stents, FFR method is the superior strategy and can be applied as a diagnostic method with high priority compared with angiography. Based on the results of the present study it seems that the use of FFR in patients with coronary stenosis may reduce cost and increase QALYs compared to the angiography. Therefore, it is suggested that considering the patient’s condition, FFR diagnostic method be used as a more cost-effective and accurate method to reduce the disease and financial burden of the disease in the community. It is also recommended improving coverage for basic and complementary insurance to treat the patients using this treatment.

This applies not only to physicians in training, but also the health policy makers use for education and continuous improvement across the span of cardiovascular diagnosticmethods.

## Supplementary Information

**Additional file 1: **Appendix CHEERS Checklist.

## Data Availability

Datasets analysed during the current study are available from the corresponding author on reasonable request.

## Abbreviations

FFR: Fractional flow reserve
ICER: Incremental cost-effectiveness ratio
PSA: Probabilistic sensitivity analysis
CAD: coronary artery stenosis
DMC: Direct medical costs
QALY: Quality adjusted life years
PPP: Purchasing power parity
PCI: Percutaneous coronary intervention
CABG: Coronary artery bypass surgery
MACE: Major adverse cardiac events
